# Seasonal patterns of vegetation drought resilience and vegetation loss in Central Asia

**DOI:** 10.1371/journal.pone.0352937

**Published:** 2026-07-02

**Authors:** Liangliang Jiang, Guangming Wu, Xinyuan Gui, Xiaoran Liu

**Affiliations:** 1 Chongqing Institute of Meteorological Sciences, Chongqing, China; 2 School of Geography and Tourism, Chongqing Normal University, Chongqing, China; 3 Chongqing Key Laboratory of GIS Application, Chongqing, China; National Cheng Kung University, TAIWAN

## Abstract

Drought events have become increasingly common in Central Asia, increasing the risk of vegetation degradation. In this study, the resilience of vegetation to drought and its drivers was investigated across different seasons. The findings revealed that western Central Asia faced a notably high incidence of spring droughts, characterized by longer durations and greater severity than droughts in other seasons. In contrast, the Aral Sea Basin experienced fewer droughts in summer and autumn, although these droughts were more severe and intense and had longer durations. Croplands, particularly those in northern Kazakhstan, generally demonstrated relatively low resistance but relatively strong resilience. In contrast, sparsely vegetated areas in regions such as southern Xinjiang and areas downstream of the Aral Sea Basin presented high drought resistance but relatively low resilience. Precipitation and vapour pressure deficit (VPD) had the most significant impact on drought resilience in Central Asia, with a combined contribution of 55.18%, particularly in the northern and eastern regions of the area. The vegetation in spring was characterized by the highest resistance and resilience levels (40.67% and 40.65%, respectively) in Central Asia, followed by those in summer. In terms of vegetation loss, vegetation in spring accounted for the greatest proportion (44.03%), followed by that in summer, at 31.07%. The main characteristics of drought (duration and intensity) were the major factors influencing the loss of vegetation, especially in grasslands and sparsely vegetated areas. During prolonged summer droughts (>40 months), grasslands and sparse vegetation suffered substantial declines in gross primary productivity (GPP). In contrast, forests exhibited more severe GPP reduction at a drought peak of around 2 and intensity above 1.5. Quantifying the resilience and loss of vegetation to drought across different seasons can aid in the formulation of effective strategies to prevent and manage vegetation degradation in Central Asia.

## 1. Introduction

Owing to global warming, extreme drought events are occurring more frequently, with heightened intensity, extended duration, and more severe consequences [1]. Extreme drought events are emerging as among the most significant threats posed by global warming, with sustained future warming expected to further increase their frequency and severity [2]. Extreme droughts have led to a reduction in the gross primary productivity (GPP) of global vegetation. The total decline in GPP caused by extreme droughts is predicted to increase from 28% from 1850–1999 to 49% from 2075–2099 under the representative concentration pathway (RCP) 4.5 scenario and 56% under the RCP8.5 scenario [3]. The continued intensification of droughts poses a threat of irreversible loss to vegetation ecosystem productivity [4,5]. Furthermore, over the past 50 years, the difference in precipitation between the rainy and dry seasons in future drought-prone areas will become increasingly pronounced, indicating that the occurrence of seasonal drought will significantly increase [6]. The risk of vegetation degradation will further increase in the region [7]. In arid regions, vegetation varies considerably throughout different seasons and years. Relying solely on short-term changes to assess the actual conditions of vegetation may result in inaccurate assessments [8,9]. Therefore, understanding how vegetation consistently responds to droughts is important, especially in relation to its resistance, recovery and drivers in Central Asia.

The response of vegetation resilience to drought events (drought resilience) is a key focal point in the current research [10,11]. The self-regulating ability of vegetation is referred to as drought resilience. However, if droughts surpass this capacity, the vegetation might not return to its original state and could deteriorate. Drought resilience can be used to reveal the self-regulation process of vegetation under external disturbances, including both the resistance phase and the recovery phase [5,12]. Drought resilience is a critical process that cannot be overlooked when vegetation changes are monitored during drought events. Furthermore, gaining insight into the dynamics of vegetation change through a resilience lens and assessing its drivers is crucial for enhancing our understanding of degradation mechanisms and for increasing the efficacy of prevention and intervention strategies [13]. The increased frequency and intensity of extreme droughts can lead to a decline in the self-recovery capacity of vegetation, resulting in irreversible degradation [14]. Considering the interaction between drought resilience and vegetation degradation, accurate determination of vegetation degradation from the perspective of resilience is critical [15].

Conventional approaches to evaluating vegetation responses to droughts involve techniques such as analysis of the changing trends and correlations, residual analysis and wavelet analysis. These methods have yielded significant findings and enhanced our understanding of how vegetation changes and responds to drought conditions [16,17]. They have been utilized to investigate the response of vegetation to droughts over the entire period or within shorter time intervals in the year of change [18]. However, vegetation in arid zones is prone to change during drought periods [19]. It is essential to account for vegetation resilience both during and after droughts and to assess periods of drought resistance and recovery [10]. Drought events can currently be identified using run theory, which can be used to determine their fundamental characteristics, such as the start and end of a drought, drought intensity, and drought interval [20]. Recognizing the significance of these traits in vegetation dynamics is essential for understanding how drought events impact vegetation [9,21]. Furthermore, the response of drought resilience to the effects of drought events is a key process in monitoring vegetation changes. In previous studies, autoregressive models were used to assess drought resilience in the context of short-term climate anomalies, thereby enhancing the quantitative analysis of drought resilience [22]. However, autoregressive models cannot be used to fully assess drought resilience to specific drought events. Instead, evaluating drought resilience involves examining the condition of the vegetation both prior to and following drought occurrence [23]. The processes of the resistance and recovery of vegetation can be quantified during particular drought events. Moreover, surface soil can retain moisture from previous rainfall events, which is a function that is vital for sustaining vegetation growth [24]. Thus, it is important to select the optimal time scale when assessing the impact of drought events on vegetation.

Additionally, the recovery status of vegetation following drought events should be considered. The recovery conditions of vegetation are related to drought resilience [25]. The extended recovery time of vegetation, associated with reduced resilience, leads to a gradual decline in productivity, posing a risk of permanent loss [3,26]. It is essential to consider the recovery conditions of vegetation after drought disturbance and examine whether they have returned to their previous state. Furthermore, drought events occurring in different seasons have varying effects on vegetation [27]. Analysing shifts in vegetation during and following drought events, particularly concerning resistance and resilience, is essential for gaining insights into how vegetation responds to droughts across various seasons [28]. Climate change has a significant effect on vegetation dynamics, yet the potential drivers influencing drought resilience are often overlooked. Considering these drivers and how they shape seasonal and regional responses offers valuable insights into the mechanisms of vegetation recovery [29]. Therefore, in this study, the resilience of vegetation across seasons was assessed, its potential drivers were identified, and vegetation loss due to drought events in Central Asia was quantified.

Frequent and severe drought events, along with increasing vegetation degradation in the arid regions of Central Asia, present significant challenges for achieving the land degradation neutrality (LDN) target by 2030 [30]. Drought resilience plays a critical role in identifying the self-regulatory mechanisms of vegetation in response to drought conditions [8]. A comprehensive understanding of how vegetation productivity is restored is crucial for the establishment of effective management strategies that counteract land degradation [31]. Nevertheless, the resilience of vegetation productivity, vegetation loss due to drought events and the corresponding drivers across various seasons are not yet fully understood. Therefore, the objectives of this study were to (1) assess drought characteristics for events occurring across various seasons, (2) quantify drought resilience in different seasons, and (3) investigate vegetation loss and its drivers in different seasons. This study provides a scientific foundation for achieving regional LDN targets.

## 2. Materials and methods

### 2.1. Study area

**Fig 1:**
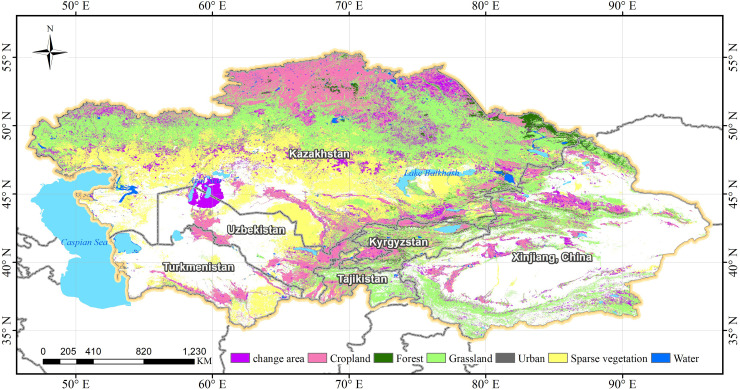
Different land cover types in Central Asia.

Central Asia encompasses the five former Soviet republics and Xinjiang Province in Central Asia, which together form a central geographical landscape characterized by a similar climate (Fig 1). The region experiences a predominantly continental climate characterized by significant temperature variations between seasons, low annual precipitation, and diverse microclimates shaped by the region’s varied topography. The arid and semiarid climate leads to low precipitation, particularly in desert areas, whereas mountainous regions experience more varied weather patterns, including higher precipitation levels and cooler temperatures [32]. These climatic conditions play crucial roles in shaping ecological and environmental patterns across the region. The distribution of vegetation in the area is shaped by its diverse climatic zones, topography, and soil conditions. The region spans vast deserts, expansive steppes, and towering mountain ranges, each supporting distinct vegetation communities. Sparse vegetation is found predominantly in southern Kazakhstan, particularly in the areas surrounding the Karakum and Kyzylkum Deserts (Fig 1). The mountainous regions feature clear vertical distributions of various forests and grasslands. Croplands are distributed mainly in northern Kazakhstan. Croplands are clearly found along rivers, whereas they are scattered across desert areas. These regions are well known for their widespread irrigated agriculture. Central Asia has experienced frequent and severe drought events [33]. Furthermore, the vegetation ecosystems in the region are extremely fragile and face severe degradation. The increasing frequency and intensity of droughts in Central Asia have negatively affected vegetation, hindering the target of zero-land degradation in the region [30]. Thus, measuring drought resilience and loss and assessing their drivers can support the development of effective strategies to prevent and manage vegetation degradation in Central Asia.

### 2.2. Data sources

In the study, various data were employed to examine drought conditions and vegetation productivity. Specifically, the ERA5-Land dataset provides monthly precipitation, temperature, solar radiation and soil moisture (SM) data for 1982–2018 at a spatial resolution of 0.1°, making it highly suitable for both regional and global climate studies [34]. Derived from the ERA5-Land dataset, the potential evapotranspiration (PET) dataset was established by using the standardized FAO method to estimate water loss through evaporation and transpiration [35]. In combination, the PET and precipitation datasets were used to compute the standardized precipitation‒evapotranspiration index (SPEI), which is a crucial indicator for evaluating drought conditions [36]. The monthly temperature, solar radiation and soil moisture (SM) data were sourced from the ERA5-Land dataset to investigate the factors influencing drought resilience in this study. ERA5-Land is widely used in environmental monitoring because of its high temporal and spatial resolutions, making it a key resource for studying climate variability, land‒atmosphere interactions, and ecosystem responses.

For vegetation productivity, we relied on the Global Land Surface Satellite (GLASS) dataset for gross primary productivity (GPP) information, covering 1982–2018, with a spatial resolution of 0.05°. This dataset integrates critical environmental parameters, such as atmospheric CO₂, radiation, and vapour pressure deficit [37]. The revised eddy covariance-light use efficiency (EC-LUE) model was used to refine the temporal resolution to eight days, with the algorithm validated against global field data from 155 sites across diverse ecosystems. The GLASS GPP dataset is extensively utilized in climate, ecological, and agricultural studies to examine vegetation productivity, carbon cycles, and the effects of environmental changes on ecosystems [37].

To incorporate land cover dynamics, the Climate Change Initiative (CCI) land use dataset was employed. This dataset provides global land cover data at a 300 m resolution obtained from satellite observations and by tracking land use changes over time [38]. This dataset offers a comprehensive view of land use categories, such as grasslands, agricultural land, forests, and water bodies. It is produced with high accuracy and a high spatial resolution, supporting research on climate change, land use planning, environmental monitoring, and biodiversity conservation [38]. According the previous studies [39,40], high-resolution remote sensing datasets were spatially resampled to a coarser resolution, and the derived outputs of the downscaled data maintain accuracy within a reasonable tolerance range. Thus, the GPP and land use data were resampled using bilinear interpolation to align with the 0.1° spatial resolution of the ERA5-Land dataset, enabling integrated analysis across the different data sources.

### 2.3. Methods

#### 2.3.1. Drought event characteristics.

**Fig 2:**
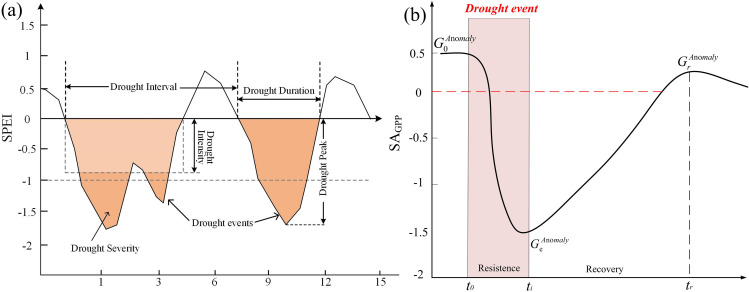
Drought events and their underlying characteristics (a) and the drought resilience process (b).

The SPEI was computed via a water balance method, making it suitable for assessing drought occurrence and severity over various time scales. However, the variability of drought effects across regions indicated that a fixed time scale for the SPEI was insufficient for comprehensive monitoring [41]. To increase the accuracy of assessing the effects of drought on vegetation, the optimal SPEI time scale was chosen on the basis of the highest correlation with the GPP, which represented the period during which vegetation was most sensitive to drought [42]. Following the established methods, the SPEI was computed using the SPEI package in R. For each pixel, the ideal time scale was identified by correlating the SPEI with the GPP, capturing the influence of drought on vegetation. Based on the run theory, a drought event was defined by two criteria: a SPEI value less than zero for more than three consecutive months and a minimum SPEI value less than −1 [43,44]. Fig 2a illustrates the primary characteristics of drought events. According to previous studies [45,46], the vegetation growing season in Central Asia spans from April to October. This period is typically divided into three parts: spring (April–May), summer (June–August), and autumn (September–October). Since drought events can impact vegetation differently depending on the season [27], they were classified according to the season in which they began.

The negative-run method was applied in each grid cell to detect drought event occurrence. Drought intensity was calculated as the ratio of drought severity to duration. The drought peak was defined as the lowest SPEI value within each negative run. The duration is the length of the drought event, while the interval indicates the time between consecutive events. Finally, the drought frequency was used to measure the number of droughts recorded over the study period. The drought characteristics were calculated as follows [43]:


$$\begin{array}{c} DS=\sum_{i=\text{1}}^{DD}\left|{SPEI}_{i}\right| \end{array}$$ (1)

$$\begin{array}{c} DI=\frac{\sum_{i=\text{1}}^{DD}\left|SPEI_{i}\right|}{DD} \end{array}$$ (2)

$$\begin{array}{c} DP=\text{max}(\left|SPEI_{i}\right|) \end{array}$$ (3)

where *SPEI*_*i*_ denotes the monthly SPEI value of the ith month. *DI* and *DD* refer to the drought intensity and drought duration, respectively. *DS* and *DP* represent the drought severity and drought peak, respectively.

#### 2.3.2. Drought resistance and resilience in vegetation.

A resilience-focused approach for evaluating the ability of vegetation to respond to and recover from drought is crucial for reducing ecosystem degradation and supporting the development of effective adaptation strategies [13]. In this study, drought resistance and resilience in vegetation are examined within a framework that uses resistance‒resilience metrics, offering insights into how vegetation adapts to drought-related disturbances (Fig 2b). To evaluate these characteristics, we calculated the standardized anomalies of monthly GPP during drought periods and subsequent vegetation recovery [47]. Drought resistance was quantified as the inverse of the percentage reduction in GPP, whereas resilience was evaluated by monitoring the change in GPP anomalies from drought conditions to the recovery phase [47]. The standardized anomalies of monthly GPP were computed using the following formula [48]:


$$\begin{array}{c} {SA}_{i}=\frac{x_{i}-\mu (x)}{\delta (x)} \end{array}$$ (4)

where ${SA}_{i}$ represents the standardized anomaly for the ith month, $x_{i}$ is the indicator value for that month, δ(x) is the standard deviation over this period, and μ(x) is the average GPP value.

Drought resistance is dynamically influenced by predrought conditions, with shifts in standardized anomalies before and after drought, providing critical insights (Fig 2b). The drought resistance was calculated via the following formula [23]:


$$\begin{array}{c} Resistance=\frac{\text{1}}{\left|\overset{Anomaly}{\underset{e}{G}}-\overset{Anomaly}{\underset{\text{0}}{G}}\right|} \end{array}$$ (5)

$$\begin{array}{c} \overset{Anomaly}{\underset{\text{0}}{G}}=\frac{\overset{Anomaly}{\underset{onset}{G}}+\overset{Anomaly}{\underset{onset-\text{1}}{G}}}{\text{2}} \end{array}$$ (6)

where *Resistance* represents drought resistance. $\overset{Anomaly}{\underset{e}{G}}$ signifies the anomalous productivity of vegetation during the month with the most intense drought, whereas $\overset{Anomaly}{\underset{\text{0}}{G}}$ indicates the anomalous productivity prior to drought onset (Fig 2b). Similarly, $\overset{Anomaly}{\underset{onset}{G}}$ represents the anomalous productivity at the beginning of the drought, and $\overset{Anomaly}{\underset{onset-\text{1}}{G}}$ corresponds to the anomaly observed in the month immediately before the drought started.

Drought resilience, defined by relative anomalies throughout and following drought events, signifies the recovery capability (Fig 2b). Drought resilience is quantified by the following formula [23]:


$$\begin{array}{c} \text{Resilience }=\left|\overset{Anomaly}{\underset{e}{G}}-\overset{Anomaly}{\underset{r}{G}}\right| \end{array}$$ (7)

where *Resilience* represents drought resilience. $\overset{Anomaly}{\underset{r}{G}}$refers to the standardized anomaly of the GPP during the month of optimal recovery. $\overset{Anomaly}{\underset{e}{G}}$ signifies the anomalous productivity of vegetation during the month with the most intense drought (Fig 2b).

When multiple drought events were detected throughout the study period; the following calculations were applied to assess the average levels of resistance and resilience.


$$\begin{array}{c} Resistance=\frac{\text{1}}{\left|\sum \overset{Anomaly}{\underset{e}{G}}-\sum \overset{Anomaly}{\underset{\text{0}}{G}}\right|} \end{array}$$ (8)

$$\begin{array}{c} \text{Resilience }=\left|\sum \overset{Anomaly}{\underset{e}{G}}-\sum \overset{Anomaly}{\underset{r}{G}}\right| \end{array}$$ (9)

Resistance indicates how well vegetation can endure drought, with higher values indicating greater drought tolerance. Resilience measures the ability of vegetation to bounce back and exceed its usual productivity afte drought. The high resistance and resilience of vegetation are indicative of superior drought endurance and recovery capabilities.

#### 2.3.3. Analysis of the factors influencing drought resilience.

Exploring the drivers of drought resilience during restoration in Central Asia provides insights into the multifaceted role of vegetation recovery after drought events, enhancing our understanding of the underlying processes involved. In this study, changes in drought resilience in relation to climatic variables were examined, with a focus on soil moisture, vapour pressure deficit (VPD), precipitation, temperature, and solar radiation. These factors were analysed to estimate their relative importance in influencing vegetation [16,49]. The selected climatic variables—VPD, precipitation, temperature, and solar radiation—along with soil moisture data were extracted to explore the drivers during the recovery period for subsequent analysis. The degree to which driving factors (E) influence drought resilience was calculated as follows [50]:


$$\begin{array}{c} E=\frac{\overset{―}{\overset{―}{D}}}{\overset{―}{\overset{―}{R}}}=\frac{\sum \left(D_{j}-\overset{―}{\overset{―}{D}}\right)\left(R_{j}-\overset{―}{\overset{―}{R}}\right)}{\sum \left(D_{j}-\overset{―}{\overset{―}{D}}\right)} \end{array}$$ (10)

where *E* refers a driving factor. j denotes the j-th year, $D_{j}$ denotes the driving factor in that year, $R_{j}$ denotes resilience, and $\overset{―}{\overset{―}{D}}$ and $\overset{―}{\overset{―}{R}}$ denote the multiyear averages of the driving factors and resilience, respectively.

The contribution rate (C) of a driving factor to drought resilience can be expressed as follows (Yin et al., 2010):


$$\begin{array}{c} C=\frac{\Delta D}{\overset{―}{\overset{―}{D}}}\times E\times \text{100}\% \end{array}$$ (11)

where ΔD denotes the variation in the driving factors. $\overset{―}{\overset{―}{D}}$ denotes the multiyear averages of the driving factors. C denotes the relative influence of these factors during the recovery period, with the largest C value indicating the key driver.

## 3. Results

### 3.1. Assessing drought characteristics in different seasons

**Fig 3:**
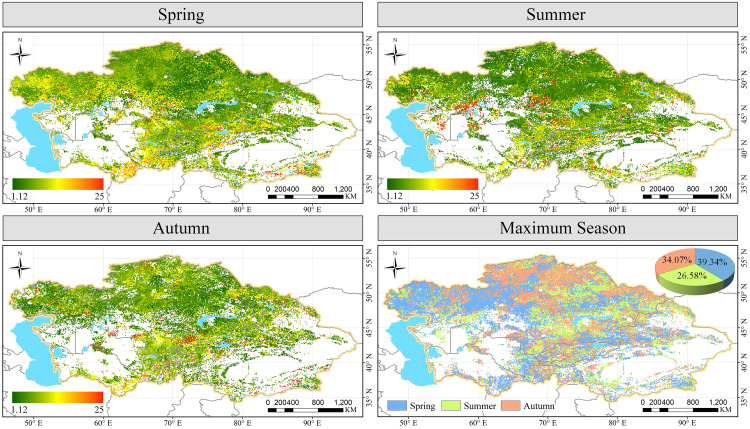
Drought severity in different seasons.

The features of drought events were investigated in Fig 3. The northern region of Central Asia presented lower drought severity in the spring, whereas the southern region presented higher drought intensity. In summer, the regions bordering the Aral Sea experienced greater drought severity than they did in spring. However, the drought severity in northern Central Asia was less severe than it was in spring. In autumn, the drought severity in northern Central Asia was greater than that in spring and summer, whereas southern Central Asia presented lower drought severity. High values of drought severity in summer and autumn were clearly concentrated in the Amu Darya Delta. Fig 3 compares the drought severity for each pixel across different seasons, with the highest values occurring in spring in 39.34% of cases, with the corresponding areas located primarily in western Central Asia.

**Fig 4:**
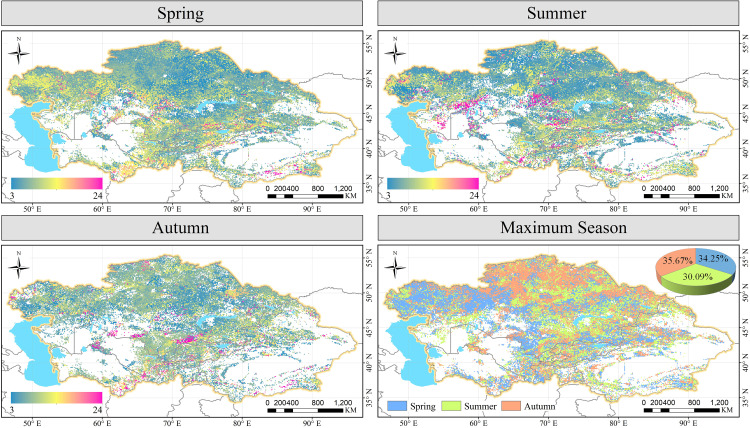
Average drought duration in different seasons.

With respect to drought duration (Fig 4), longer values in spring were observed in southern Central Asia, whereas shorter values were observed in northern Central Asia. In summer, the duration of drought in the western region was shorter than that in spring. However, the central area of Central Asia experienced long drought durations. In autumn, the duration of drought in northern Central Asia was longer than that in spring and summer, whereas in western Central Asia, the duration of drought events was short. Notably, areas such as the Amu Darya Delta were significantly impacted and experienced droughts that lasted approximately 24 months. The longest drought duration occurred in autumn, accounting for 35.67% of the total drought duration, followed by spring, at 34.25%.

The spatial distribution of drought intensity is displayed in S2 Fig. High values in spring were observed across most areas, particularly in northern Kazakhstan and the upstream area of the Aral Sea Basin. In summer, the drought intensity decreased in most regions. Evidently, the drought intensity in northern Kazakhstan and the upstream region of the Aral Sea Basin was lower than that in spring. The drought intensity in autumn increased in some regions, such as the Syr Darya River Basin. The highest drought intensity occurred in spring, accounting for 51.14% of the total, followed by summer, at 24.50%. With respect to the drought peak (S3 Fig), 39.41% of Central Asia presented a greater drought peak in spring than in summer and autumn, with these areas mainly concentrated in Kazakhstan. The drought peak in summer decreased in most regions. Notably, the drought peaks in the Syr Darya River Basin and western Kazakhstan were relatively high in autumn.

Most drought events in Central Asia occurred in spring (S4 Fig). Specifically, 67.21% of the study area experienced more drought in spring than in summer and autumn, with these areas being primarily concentrated in the western region. Eastern Kazakhstan experienced more drought in summer. In contrast, the duration of drought in autumn was relatively short in most areas. With respect to the drought interval (S5 Fig), the majority of regions experienced relatively brief drought intervals in spring, suggesting an increased occurrence of drought events in spring. In contrast, the upstream region of the Aral Sea Basin experienced a lower frequency of drought events in summer. In autumn, Xinjiang Province in Central Asia experienced a relatively long drought interval, suggesting that drought events occurred infrequently in this area. Overall, western Central Asia experienced drought events with greater frequency, longer durations and greater severity in spring than in the other seasons. In contrast, the Aral Sea Basin experienced fewer droughts during the summer and autumn; however, these events were characterized by significant severity, intensity, and durations.

### 3.2. Quantifying drought resilience and its drivers in different seasons

**Fig 5:**
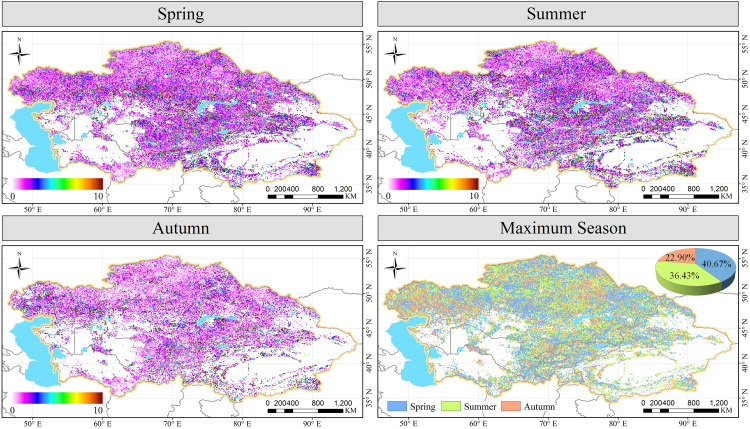
Spatial patterns of drought resistance during different seasons.

Drought resistance in vegetation was evaluated across various seasons, as illustrated in Fig 5. In spring, the resistance of vegetation in northern Kazakhstan was lower than that in other regions. In contrast, the vegetation resistance was high in the Tianshan, Pamirs and central regions of Central Asia. The vegetation surrounding Lake Balkash exhibited high resistance. In summer, the vegetation in eastern Kazakhstan was more resistant than it was in spring, especially in northeastern Kazakhstan and the area surrounding Lake Balkash. However, the drought resistance in mountainous areas was lower than that in spring. In summer, the vegetation resistance of the Amu Darya Basin, particularly in the delta region, significantly decreased. The vegetation in autumn exhibited low resistance in most areas. Furthermore, low drought resistance was concentrated in northern Kazakhstan. Drought resistance was compared across different seasons in Fig 5. Compared with those in the other seasons, 40.67% of the vegetation in Central Asia presented the highest resistance in spring. The highest drought resistance in summer and autumn accounted for 36.43% and 22.90%, respectively. Overall, the vegetation in most regions exhibited lower drought resistance in autumn than in other seasons.

**Fig 6:**
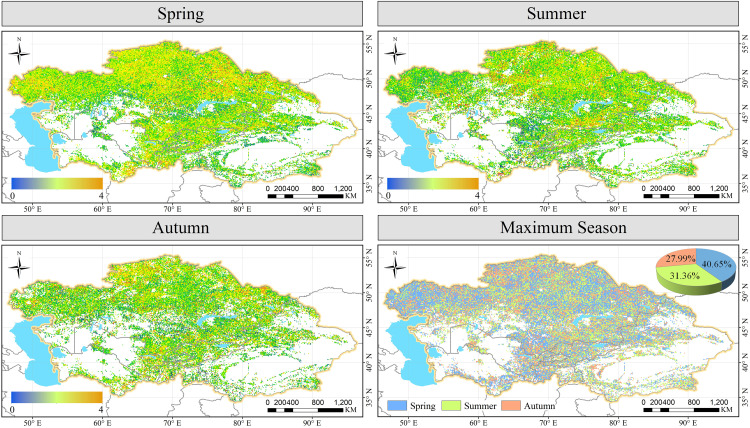
Spatial patterns of drought resilience across different seasons.

Fig 6 presents the drought resilience across different seasons. During spring, vegetation in most of northern Kazakhstan demonstrated considerable resilience, whereas vegetation in southern Central Asia exhibited low resilience, especially in southern Xinjiang and downstream of the Aral Sea Basin. The spatial distribution of drought resilience in summer was similar to that in spring. However, the drought resistance in most regions was lower than that in spring, particularly in northern Kazakhstan and the Aral Sea Basin. In autumn, vegetation exhibited low resilience in most areas. Furthermore, the areas with high drought resilience in northern Kazakhstan were further reduced. Throughout the entire vegetation area, 40.65% of the vegetation in spring presented the highest resilience compared with that in the other seasons, followed by that in summer (31.36%). The highest degree of drought resistance, 27.99%, occurred in autumn. Overall, the vegetation in most regions exhibited greater drought resilience in spring than in summer and autumn.

**Fig 7:**
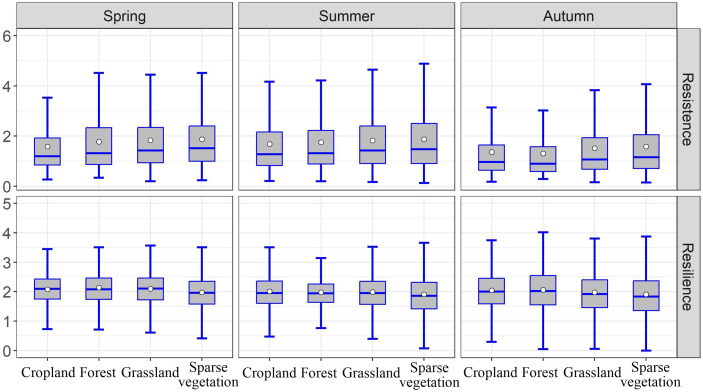
Drought resistance and resilience in vegetation across various land cover types. The solid dots in each box represent the average values for different land cover types.

Fig 7 presents a comparison of the levels of drought resistance and resilience across various land cover types. Among the various types of land cover, sparse vegetation presented the highest level of resistance to drought. The average drought resistance values increased in spring and summer for croplands, forests, grasslands, and sparse vegetation. In autumn, the resistance of croplands and grasslands was not significantly different. Compared with that in the other seasons, the drought resistance in cropland and sparse vegetation areas was greater in summer than in spring, whereas the drought resistance in forests was lower in summer than in spring. The drought resistance across all the land cover types was greater in spring and summer than in autumn. With respect to drought resilience (Fig 7), compared with other land cover types, sparse vegetation exhibited lower resilience. Among the different land covers, croplands and grasslands presented the greatest drought resilience. Among the different seasons, grasslands and sparse vegetation exhibited greater resistance in spring than during the summer and autumn months, whereas forest resilience was notably lower in summer than in both spring and autumn. In general, croplands presented low drought resistance and high drought resilience. Conversely, sparse vegetation had high resistance and low resilience. The drought resistance was greater in spring and summer than in autumn, whereas the drought resilience was greater in autumn than in spring and summer.

**Fig 8:**
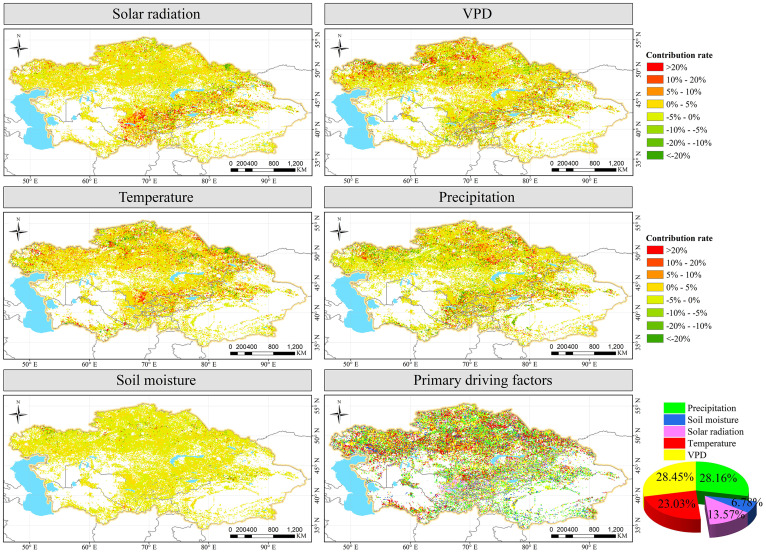
Spatial distributions of the importance of different driving factors in determining the contribution of drought resilience in autumn.

Fig 8, S6 and S7 Figs illustrate the seasonal variations in the primary drivers of drought resilience across Central Asia. Spring vegetation recovery was influenced predominantly by precipitation (27.55%), VPD (24.93%), and temperature (23.3%), especially in the northern areas. In contrast, the influence of solar radiation was relatively minor (13.07%), with areas with high contribution rates (>20%) distributed mainly in the eastern part. In summer, the influence of temperature-related factors on vegetation tended to decrease, whereas the rates of influence of precipitation and VPD increased by 1.69% and 0.67%, respectively. This pattern was particularly pronounced in the western and northern regions. In autumn, precipitation, VPD, and temperature remained the dominant factors, with the rates of influence of temperature and VPD significantly increasing compared with those in summer, especially in the northern and eastern areas. Notably, the VPD increased by 2.85% in autumn, making it the most influential factor driving vegetation recovery. Throughout the growing season, precipitation, VPD, and temperature were the primary factors regulating vegetation recovery in Central Asia. Notably, the average contribution of precipitation reached 28.85% during spring and summer, underscoring its critical role in supporting vegetation recovery under drought conditions. However, the influence of the VPD continuously increased, reaching 28.45% in autumn and surpassed that of precipitation as the dominant factor driving vegetation recovery.

### 3.3. Evaluating vegetation loss during drought events in different seasons

**Fig 9:**
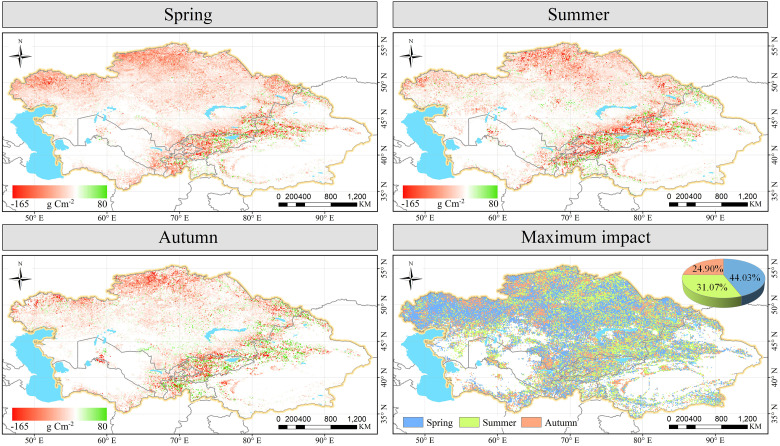
Vegetation loss during drought events across different seasons.

The most significant impacts of drought events on vegetation were extracted at the pixel level over the study period. Vegetation loss during the greatest impact of drought events was investigated in different seasons (Fig 9). In spring, a high reduction in GPP (−165 g C m^-2^) was observed in mountainous regions, including Tianshan and Pamirs. A low reduction in GPP was detected in central Kazakhstan. The decrease in GPP was relatively concentrated in western and northern Kazakhstan. In summer, the decrease in GPP further intensified in northern Kazakhstan and mountainous regions. However, the decrease in GPP was alleviated in western and central Kazakhstan. The reduction in GPP in most regions was lower in autumn than in summer. Notably, the Amu Darya delta experienced a high reduction in GPP in autumn. The increase in GPP in autumn was greater than that in the previous two seasons. Throughout the entire vegetation area, 44.03% of the vegetation in spring presented greater vegetation loss than that in the other seasons did, followed by that in summer (31.07%). The greatest vegetation loss in autumn accounted for 24.09% of the total loss. Overall, drought events in spring had the greatest impact on vegetation in Central Asia. However, vegetation loss gradually intensified in northern Kazakhstan and mountainous regions from spring to autumn.

**Fig 10:**
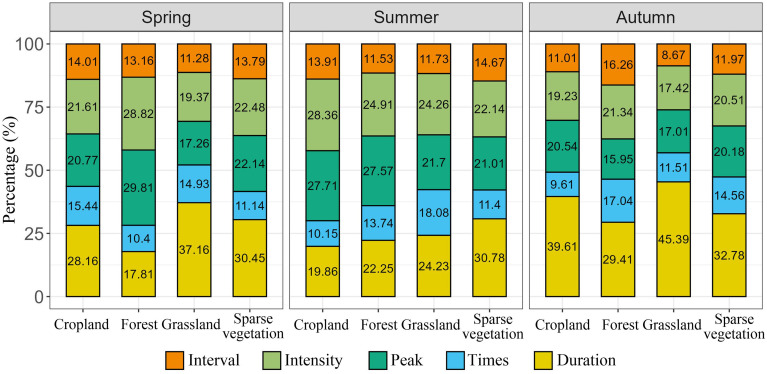
Influence of drought characteristics on vegetation changes in the Boosted Regression Tree (BRT) model.

According to the Boosted Regression Tree (BRT) model (Supplementary material), the influence of drought characteristics on vegetation loss is quantified across different seasons (Fig 10). In spring, the model demonstrated that drought duration and intensity had the most significant impact on vegetation loss in cropland, grassland and sparse vegetation, areas accounting for approximately 60% of the overall impact. In forests, vegetation loss was most highly related to the drought peak (29.81%) and drought intensity (28.82%). In summer, drought duration and intensity were the main factors affecting vegetation loss in grasslands and sparse vegetation, whereas drought peak and intensity had the most significant relations with vegetation loss in croplands and forests. In autumn, drought duration had the greatest effect on GPP reduction across various land cover types, contributing to more than 30% of the total impact. The effect of drought intensity on GPP decreased across all land cover types, accounting for approximately 20% of the total impact. In general, drought duration and intensity were the primary factors affecting vegetation loss during drought events across different seasons, particularly in grasslands and areas of sparse vegetation. The drought peaks in spring and summer were the main factors related to vegetation loss, especially in forests and croplands.

Two key indicators were selected to examine the impact of drought characteristics on vegetation loss during spring (S8 Fig). In spring, the vegetation reduction was greater in croplands and forests than in grasslands and sparse vegetation. The joint pairwise interaction plots for croplands revealed that vegetation experienced a significant decrease in GPP in regions where the drought duration ranged from 20 to 30 months and where the drought intensity was between 0.5 and 1. Forest areas with drought peaks ranging from 2 to 3 and drought intensities exceeding 1 experienced high reductions in GPP. Grasslands presented greater decreases in GPP in regions where the duration of drought exceeded 40 months. Sparse vegetation exhibited greater decreases in GPP in regions where the drought duration was approximately 20 months and where the drought intensity exceeded 1.5.

**Fig 11:**
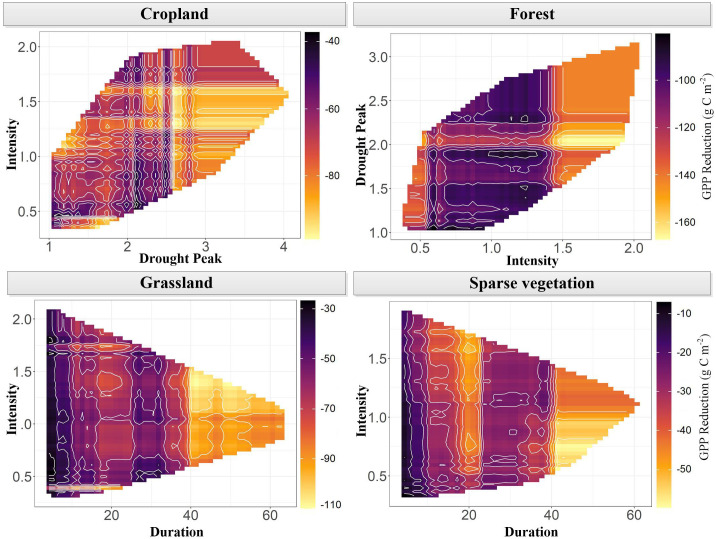
Joint pairwise interaction plots illustrating the impact of the two most significant drought features on vegetation changes in summer.

In summer (Fig 11), grasslands and sparse vegetation experienced greater decreases in GPP in areas where the drought duration exceeded 40 months. Forests experienced greater decreases in GPP in regions where the drought peak was approximately 2 and the drought intensity exceeded 1.5, whereas croplands experienced a significant decrease in GPP in regions where the drought peak exceeded 3 and where the drought intensity was between 1.25 and 1.6. In autumn (S9 Fig), vegetation areas with drought durations exceeding 40 months presented greater decreases in GPP in croplands, grasslands and sparse vegetation. Forests experienced greater decreases in GPP in areas where the drought intensity exceeded 1.25 and the drought duration exceeded 10 months. In general, as the duration of drought increased, the reduction in GPP became more pronounced across most land cover types, especially grasslands and sparse vegetation. Significant reductions in GPP in grasslands and sparse vegetation occurred in areas where the drought duration exceeded 40 months. Forests experienced greater decreases in GPP in regions where the drought intensity exceeded 1.25, especially in summer and autumn.

## 4. Discussion

### 4.1. Drought resistance and resilience in vegetation

In Central Asia, drought events occurred more frequently in spring, with higher intensity and severity than in other seasons, particularly in western Central Asia (Fig 3, S2 and S4 Fig). Spring droughts are closely linked to the East Atlantic/West Russia (EAWR) pattern [32], which is influenced by cooling in the tropical central‒eastern Pacific and is potentially linked to the impact of La Niña on precipitation in this region [51]. This EAWR pattern, characterized by northwards displacement of moisture flows [51] and diminished moisture transfer from the Persian Gulf and the Arabian Sea, contributed to reduced precipitation across Central Asia [52]. This phenomenon aligns with previous findings showing that limited precipitation and increased evapotranspiration intensify spring droughts and disrupt the water supply balance in Central Asia, exacerbating seasonal dryness [49,53]. In contrast, the Aral Sea Basin experienced fewer droughts in summer and autumn, although these events tended to be severe, prolonged, and intense (Fig 3, S2 and S4 Fig). Climatic shifts in this area, driven by the degradation of the Aral Sea and widespread land use changes, have heightened the region’s vulnerability to precipitation deficits, which is consistent with previous studies [30,54]. Future projections indicate that the Aral Sea Basin will likely face even drier conditions in the future, particularly in the middle and lower reaches [55]. These trends have compounded pressures on agriculture, water resources, livestock, and public health systems, highlighting the area’s susceptibility to climate-driven stressors [56].

The response of vegetation to drought varied across regions and seasons. Croplands upstream of the Aral Sea and in northern Kazakhstan demonstrated low resistance but high resilience, especially in spring (Figs 5 and 6). The rainfed croplands in these areas relied primarily on spring rainfall to meet the water demand. Drought in these regions has significantly affected rainfed crops [57]. Moreover, more frequent and intense drought events occurred in these regions during the spring (S2 and S4 Figs). This phenomenon will worsen the current water shortages in rainfed croplands throughout Central Asia. Moreover, rainfed croplands have high potential for rapid recovery after drought events because of rewetting [58]. The rainfed croplands exhibited greater recovery capacity and high resilience after drought events, which is consistent with previous studies [30,59]. Furthermore, rainfed wheat is widely grown in northern Kazakhstan, and compared with irrigated cotton, rainfed wheat in this area has significantly lower water requirements than irrigated cotton does, thereby increasing its ability to recover from droughts [60]. Thus, croplands in northern Kazakhstan exhibited a strong capacity for drought resilience, especially in spring (Fig 6).

In contrast, most irrigated croplands in Xinjiang Province and southern Central Asia presented high drought resistance and low resilience (Figs 5 and 6). Irrigation helps mitigate the effects of drought and improves the resistance of irrigated cropland to extreme drought conditions [61]. Consequently, the drought resistance was greater in these areas than in rainfed croplands. Furthermore, irrigated cotton served as the primary crop in these areas, demanding considerable water resources. Since the 1960s, there has been a notable increase in cotton cultivation and a corresponding increase in agricultural water usage [56]. The expansion of irrigated areas, which are not sufficiently offset by improvements in efficiency and storage, reduced their resilience to drought events [61], especially in the Aral Sea Basin. Thus, the vegetation near the Aral Sea exhibited limited resilience, particularly in the summer (Fig 6). Dai et al. demonstrated that irrigated croplands exhibit lower resilience than rainfed croplands [59].

Grasslands and sparse vegetation in the study area exhibited considerable resistance; however, their resilience remained low, particularly in summer (Fig 7). Grasslands and sparse vegetation are primarily found in southern Central Asia. This region is characterized by arid and hyperarid conditions, featuring stress-resistant natural vegetation that uses various strategies to endure drought events, such as strategies that include tapping into moisture from deeper soil layers [62]. Furthermore, the roots of sparse vegetation in arid areas extend deeper into the soil than the roots of grasslands plants do, enabling them to withstand drought conditions more effectively and access water from greater depths [49]. Thus, compared with grasslands, sparse vegetation exhibited greater resistance (Fig 7). For different seasons, the resistance of grasslands and sparse vegetation was greater in summer than in spring. The low resistance of grasslands and sparse vegetation in spring can be attributed to their low tolerance during the early growth stage [27]. Notably, the resilience of grasslands and sparse vegetation was greater in spring than in summer (Fig 7). The vegetation had a greater capacity to recover from external disturbances in spring. The lower demands for photosynthesis and nutrients in spring enabled vegetation to return to optimal growth conditions more quickly after disturbances than in summer [27]. Furthermore, the growth of grasslands and sparse vegetation in summer was partially influenced by their conditions in spring, which could lead to reduced resilience during summer [63]. Forests have shown low resistance and high resilience in Central Asia (Fig 7). According to Sun et al [64]. Forests harboring high species diversity exhibit more intricate compositional and structural characteristics than other vegetation types. Thus, forests are more resilient than other vegetation types. Mountain forests include drought-sensitive species (e.g., *Sabina przewalskii* and *Pinus sylvestris*), which reduce their leaf area to limit water loss but recover rapidly under normal moisture availability [65]. Protecting early growing-season growth is critical for these low-resistance forests.

Precipitation and VPD were the dominant drivers of drought resilience in Central Asia, jointly explaining 55.18% of the total variation (Fig 8, S6 and S7 Figs). Zhang et al revealed that vegetation resilience in GPP is highly vulnerable to drought and VPD, especially in high-altitude areas [66]. This highlights moisture as a critical regulator of vegetation productivity in arid regions, where plants exhibit heightened sensitivity to water availability [67]. Rising CO₂ levels further amplify this sensitivity by reducing plant transpiration [68]. High VPD levels accelerate the evaporation of atmospheric moisture, thus reducing the moisture available to vegetation in arid regions [66]. The lack of atmospheric moisture and precipitation during the growing season drives vegetation stomatal closure, further reducing drought resilience [29]. In humid regions, however, sufficient atmospheric moisture and precipitation satisfy the water demands for vegetation growth and resilience, thereby positively affecting photosynthesis and promoting the synthesis of vegetation productivity [41]. Thus, the contribution of vegetation productivity is greatest in regions with abundant atmospheric moisture and precipitation. Arid ecosystems, however, display a near-linear productivity response to moisture fluctuations. Decreases in precipitation or atmospheric moisture during the growing season may lead to hydraulic failure, severely impairing vegetation recovery [66].

### 4.2. Vegetation loss during drought events

Drought significantly reduced gross primary productivity (GPP) in the Tianshan and Pamirs Mountains (Fig 9), where warm, arid conditions increase water and heat stress, and suppress forest and grassland growth [69]. Droughts can decrease photosynthesis in the forest canopy and lower the water use efficiency of root systems, leading to reduced growth rates, particularly in summer [65]. Vegetation growth in the extreme environments of Tianshan and Pamirs was most significantly impacted by the most severe drought events. Compared to other areas, those at high elevations experience cooler temperatures and reduced biodiversity. These areas become unstable when disrupted by droughts and are challenging to restore after damage occurs. Li et al. also showed decreasing trends in vegetation greenness in the central Tianshan Mountains from 1982 to 2013 [70]. Droughts were responsible for triggering 72.8% of forest degradation [65]. Conversely, forests in the Altai Mountains presented an increase in GPP during drought events across different seasons (Fig 9). The Altai Mountains are characterized by cold and humid climatic conditions. Forest growth, particularly of Siberian larch trees, is constrained by low temperatures but shows increasingly positive responses to these temperatures over time [70]. Warmer springs promote cambial activation and reduce frost damage, whereas earlier snowmelt enhances water availability for seedling recruitment in water-limited regions [71]. Furthermore, higher spring temperatures reduce the freezing risk, improving seedling survival. In summer, seedlings at relatively high altitudes may be more sensitive to light availability than to drought stress [72]. However, vegetation growth in low-altitude stands consistently responded negatively to drought stress [71], with a decrease in GPP observed during drought events (Fig 9).

The GPP decreased in northern Kazakhstan during droughts, with rainfed croplands and grasslands being particularly vulnerable (Fig 9). Frequent droughts (S2 and S4 Figs) have reduced crop yields, threatening food security [73]. Additionally, grasslands in western Kazakhstan experienced a significant decrease in GPP because of low drought resistance (Figs 5 and 9). This aligns with the observed vegetation degradation in this region [30]. Droughts in western Kazakhstan were driven by temperature and tended to intensify over the past few decades. Furthermore, the regions faced soil erosion as a result of the abandonment of cultivated land [49], making vegetation more susceptible to droughts. In contrast, southern Central Asia, which has a low GPP, exhibited only a slight decline in GPP during drought events (Fig 9). However, a significant reduction in GPP was detected in the lower Amu Darya region, particularly in summer and autumn, because agricultural expansion altered water flows and intensified downstream scarcity [74]. Substantial increases in water stress and vegetation degradation were observed in this region. Furthermore, the shrinkage of the Aral Sea further exacerbated conditions, lowering groundwater by 0.15–3.39 m and stressing vegetation during dry seasons [30]. These high-degradation areas should be prioritized in restoration planning.

The main drought characteristics contributing to vegetation decline in Central Asia, particularly during the summer and autumn, were drought duration and intensity (Fig 10). As drought duration and intensity increased in dry years, the negative effects on vegetation increased, leading to a significant decline in GPP (Fig 11, S8 and S9 Figs). The duration of drought recovery is directly related to the duration of drought. That is, the longer the drought is, the more time it takes to recover from the drought, which aligns with the findings of Ahmadi et al. [75]. During prolonged drought events, the combination of high evapotranspiration demand and low soil moisture severely limits carbon absorption, which can result in the death of vegetation [76]. The prolonged vegetation response time to drought in Central Asia, which lasts more than 12 months, could be linked to how vegetation in semiarid ecosystems adapts to water shortages. The vegetation in the region has become more drought resistant and is better adapted to long response periods [77]. Thus, grasslands and sparse vegetation experienced significant GPP reductions in areas where drought persisted for more than 40 months (Fig. 11, S8 and S9 Figs). Additionally, the extent of damage varies among different vegetation types because of their distinct adaptation strategies. The likelihood and extent of triggering a decline in vegetation productivity are anticipated to increase with increasing drought intensity. Forest loss is also associated with the intensity of drought, as reported by previous studies [78,79]. Forests experienced greater decreases in GPP in regions where the drought intensity exceeded 1.25, especially in spring and autumn (S8 and S9 Figs). Therefore, the effect of drought on vegetation productivity is influenced mainly by both the severity and duration of the drought [80]. Importantly, grasslands and sparse vegetation were most vulnerable to drought duration and drought intensity (Fig 10).

## 5. Conclusions

In the study, drought resilience was quantified, and its drivers across different seasons in Central Asia were identified. Additionally, we assessed vegetation loss due to drought across different seasons and evaluated the influence of different drought characteristics on vegetation loss. The main findings are as follows:

(1) In spring, compared with other regions, western Central Asia faced more frequent droughts with longer durations and greater severity. Conversely, southern Central Asia experienced fewer drought events in summer and autumn, although these events were notably severe, intense, and prolonged. Overall, 67.21% of spring droughts in Central Asia are associated with long drought durations beyond those in summer and autumn.
(2) Croplands exhibited low drought resistance but high drought resilience, especially in northern Kazakhstan. Conversely, sparse vegetation resulted in high resistance to drought but low resilience, especially in regions such as southern Xinjiang and areas downstream of the Aral Sea Basin. Precipitation had the most significant impact on drought resilience in Central Asia, closely followed by VPD, with a combined contribution of 55.18%, particularly in the northern and eastern regions of the area. The drought resistance and resilience were greater in spring than in summer and autumn. 40.67% and 40.65% of the vegetation in Central Asia presented the highest resistance and resilience in spring, respectively, followed by summer.
(3) In Central Asia, 44.03% of the vegetation in spring presented greater loss than it did in the other seasons, followed by summer (31.07%). Vegetation loss gradually intensified in northern Kazakhstan and mountainous regions from spring to autumn. The primary drivers of vegetation loss during drought events were drought duration and intensity, particularly in grasslands and sparse vegetation. In summer, grasslands and sparse vegetation experienced greater decreases in GPP in areas where the drought duration exceeded 40 months, whereas forests experienced greater decreases in GPP in regions where the drought peak was approximately 2 and the drought intensity exceeded 1.5.

## Supporting information

S1 Text Vapour Pressure Deficit (VPD) and Boosted regression trees. (DOCX)

S1 Fig Spatial pattern of river basins in Central Asia. (TIF)

S2 Fig The average drought intensity of drought events in different seasons. (TIF)

S3 Fig The average drought peak of drought events in different seasons. (TIF)

S4 Fig The average drought time of drought events in different seasons. (TIF)

S5 Fig The average drought interval of drought events in different seasons. (TIF)

S6 Fig Spatial distribution of different driving factors importance in determining the contribution of drought resilience in spring. (JPG)

S7 Fig Spatial distribution of different driving factors importance in determining the contribution of drought resilience in summer. (JPG)

S8 Fig Joint pairwise interaction plots illustrating the impact of the two most significant drought features on vegetation change in spring. (TIF)

S9 Fig Joint pairwise interaction plots illustrating the impact of the two most significant drought features on vegetation change in autumn. (TIF)

S1 Table Collinearity test for drought characteristics. (DOCX)

S2 Table Collinearity test for drought characteristics after removing drought severity. (DOCX)
