# Supplementary material for: Seasonal patterns of vegetation drought resilience and vegetation loss in Central Asia
Source: PLoS One. 2026 Jul 2;21(7):e0352937. doi: 10.1371/journal.pone.0352937 (PMC13327245; doi:10.1371/journal.pone.0352937)
Supplement: S1 Text — (DOCX) [file pone.0352937.s001.docx]

*Supplementary Information*

“Seasonal Patterns of Vegetation Drought Resilience and Vegetation Loss in Central Asia”

Liangliang Jiang ^1,^ ^2, 3*^, Guangming Wu ^2, 3^, Xinyuan Gui ^2, 3^, Xiaoran Liu ^1^

^1^ Chongqing Institute of Meteorological Sciences, Chongqing, China

^2^ School of Geography and Tourism, Chongqing Normal University, Chongqing, China

^3^ Chongqing Key Laboratory of GIS Application, Chongqing, China

**Extended description of methods**

**Text S1: Vapour Pressure Deficit (VPD) and Boosted regression trees**

The VPD data were calculated on the Google Earth Engine (GEE) platform on the basis of elevation (Yao et al., 2024), surface temperature, and dew point temperature provided by ERA5-Land, as follows:

$$\begin{aligned} SVP=6.112\times\left( 1+7\times10-4+3.46\times10-6\times p_{\mathrm{mst}} \right)\times e^{\frac{17.67*\mathrm{Ta}}{Ta+243.5}}\#\left( 1 \right) \end{aligned}$$

$$\begin{aligned} AVP=6.112\times\left( 1+7\times10-4+3.46\times10-6\times p_{\mathrm{mst}} \right)\times e^{\frac{17.67*\mathrm{Td}}{Td+243.5}}\#\left( 2 \right) \end{aligned}$$

$$\begin{aligned} p_{\mathrm{mst}}=1013.25\times\left( \frac{273.16+Ta}{273.16+Ta+0.0065\times Z} \right)^{5.625}\#\left( 3 \right) \end{aligned}$$

$$\begin{aligned} VPD=SVP-AVP\#\left( 4 \right) \end{aligned}$$

where $p_{\mathrm{mst}}$ denotes the atmospheric pressure correction factor, and 1013.25 hPa represents the standard 、atmospheric pressure at sea level. $\mathrm{Ta}$ indicates the surface temperature of the land (°C), $\mathrm{Td}$ is sourced from the ERA5 dataset and corresponds to the dew point temperature (°C), and $Z$ represents elevation (m). The SVP and AVP represent the saturated and actual vapour pressures (kPa), respectively.

**Boosted regression trees**

In this study, the impact of various drought characteristics on vegetation loss were evaluated using the boosted regression tree (BRT) model. This model employs gradient boosting, a technique that sequentially adds trees, each aligned with the gradient, to minimize residual error (Li et al., 2020). The BRT approach is particularly robust to missing data and irrelevant predictors and recalculates residual deviance at each iteration, allowing the model complexity to increase step-by-step. After each fold, the optimal tree count is determined by minimizing the holdout deviance, with the standard error evaluated to ensure model stability (Hastie et al., 2009). Cross-validation and random subsampling are then used to prevent overfitting and improve the prediction accuracy (Naghibi et al., 2016). Owing to its robustness in handling diverse ecological predictors, the BRT model is widely employed in ecological studies. To identify the optimal configuration based on the lowest prediction error, various combinations of learning rates ranging from 0.1 to 0.0001 and tree complexities from 1 to 10 were evaluated. The results showed that a learning rate of 0.01, a tree complexity of 6, and a bag fraction of 0.5 yielded high predictive accuracy.

The variance inflation factor (VIF) approach was employed to detect potential multicollinearity among drought characteristics. Variables with VIF values exceeding 10, coupled with tolerance values less than 0.1, were considered to have significant multicollinearity, which could inflate regression coefficient variances (O’brien, 2007; Tamura et al., 2019). An initial assessment revealed high VIF values for drought severity and duration (S1 Table), indicating substantial multicollinearity; thus, drought severity was excluded, and collinearity was reassessed (S2 Table). Ultimately, five drought attributes—intensity, peak, interval, duration, and timing—were selected for analysis in the BRT model. The model was developed using the "dismo" package in R, with its parameters optimized for accurate prediction (Hijmans and Elith, 2013).

References

Hastie T, Tibshirani R, Friedman JH, Friedman JH. The elements of statistical learning: data mining, inference, and prediction. Vol 2: Springer, 2009.

Hijmans RJ, Elith J. Species distribution modeling with R. R package version 0.8-11 2013.

Li X, Piao S, Wang K, Wang X, Wang T, Ciais P, et al. Temporal trade-off between gymnosperm resistance and resilience increases forest sensitivity to extreme drought. Nature Ecology & Evolution 2020; 4: 1075-1083.

Naghibi SA, Pourghasemi HR, Dixon B. GIS-based groundwater potential mapping using boosted regression tree, classification and regression tree, and random forest machine learning models in Iran. Environmental monitoring and assessment 2016; 188: 44.

O’brien RM. A caution regarding rules of thumb for variance inflation factors. Quality & quantity 2007; 41: 673-690.

Tamura R, Kobayashi K, Takano Y, Miyashiro R, Nakata K, Matsui T. Mixed integer quadratic optimization formulations for eliminating multicollinearity based on variance inflation factor. Journal of Global Optimization 2019; 73: 431-446.

Yao Y, Liu Y, Fu F, Song J, Wang Y, Han Y, et al. Declined terrestrial ecosystem resilience. Global Change Biology 2024; 30: e17291.
