# Supplementary figures and images for: Seasonal patterns of vegetation drought resilience and vegetation loss in Central Asia
Source: PLoS One. 2026 Jul 2;21(7):e0352937. doi: 10.1371/journal.pone.0352937 (PMC13327245; doi:10.1371/journal.pone.0352937)

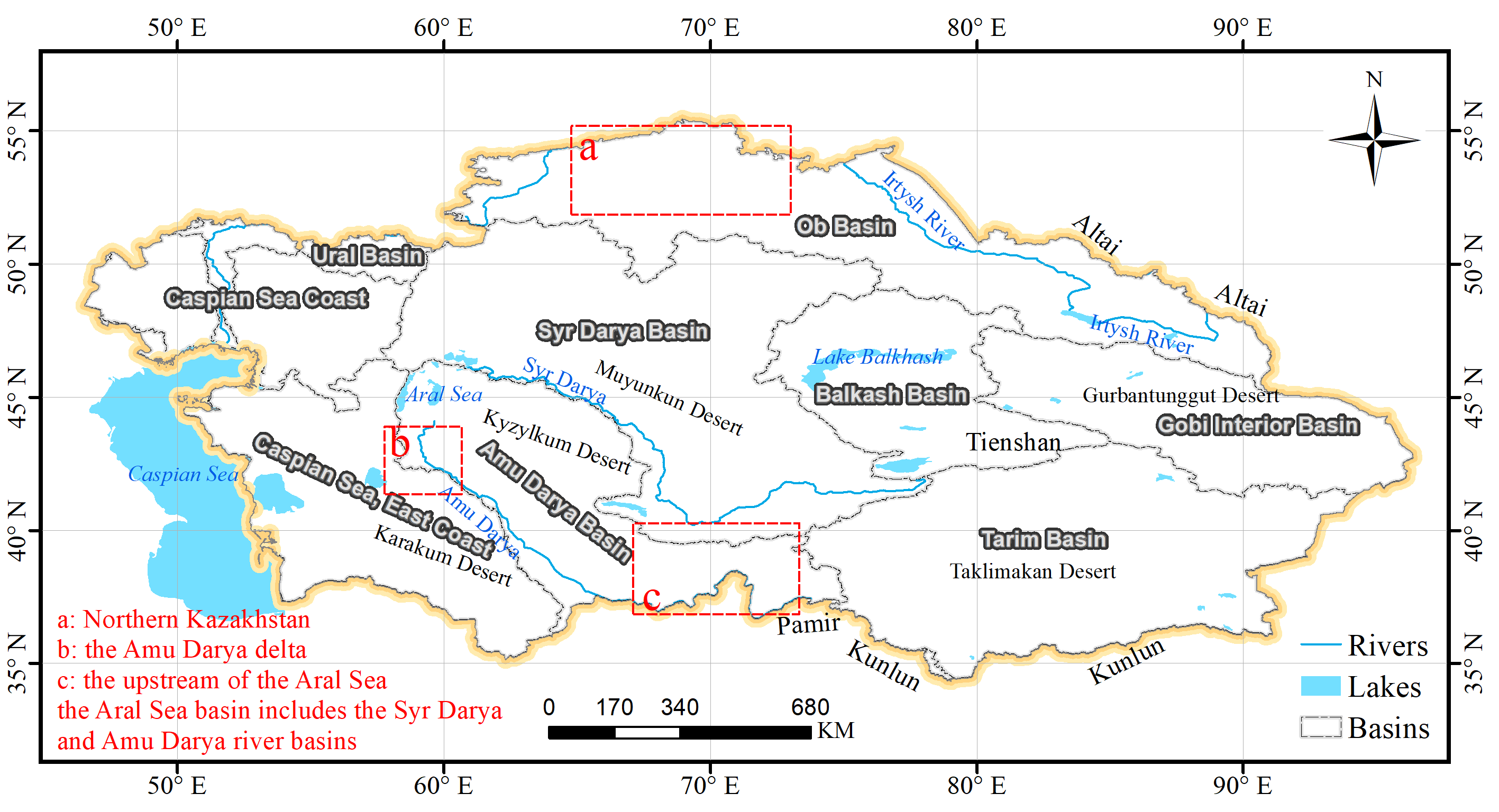

Supplement: S1 Fig — (TIF) [file pone.0352937.s002.tif]

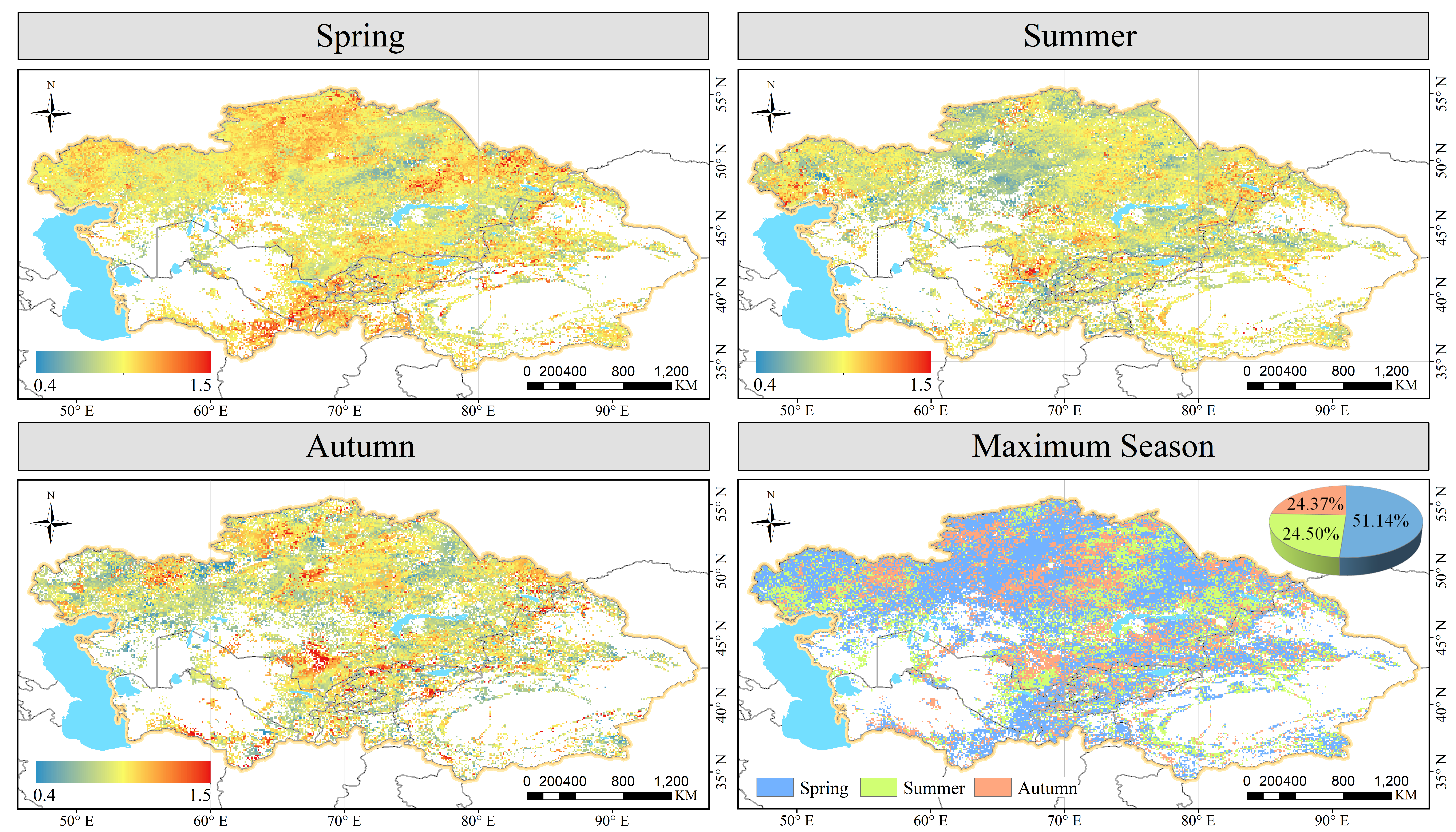

Supplement: S2 Fig — (TIF) [file pone.0352937.s003.tif]

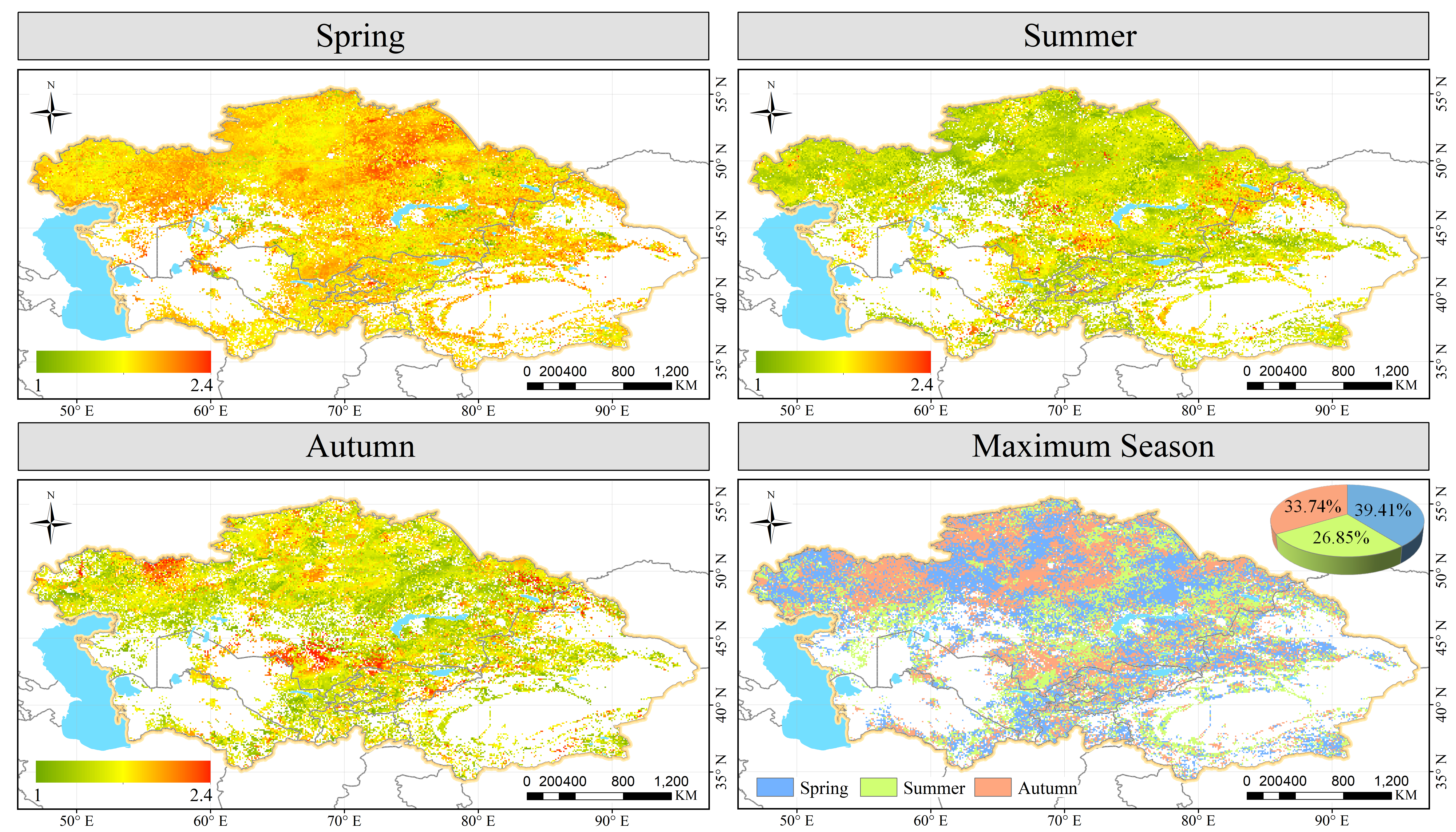

Supplement: S3 Fig — (TIF) [file pone.0352937.s004.tif]

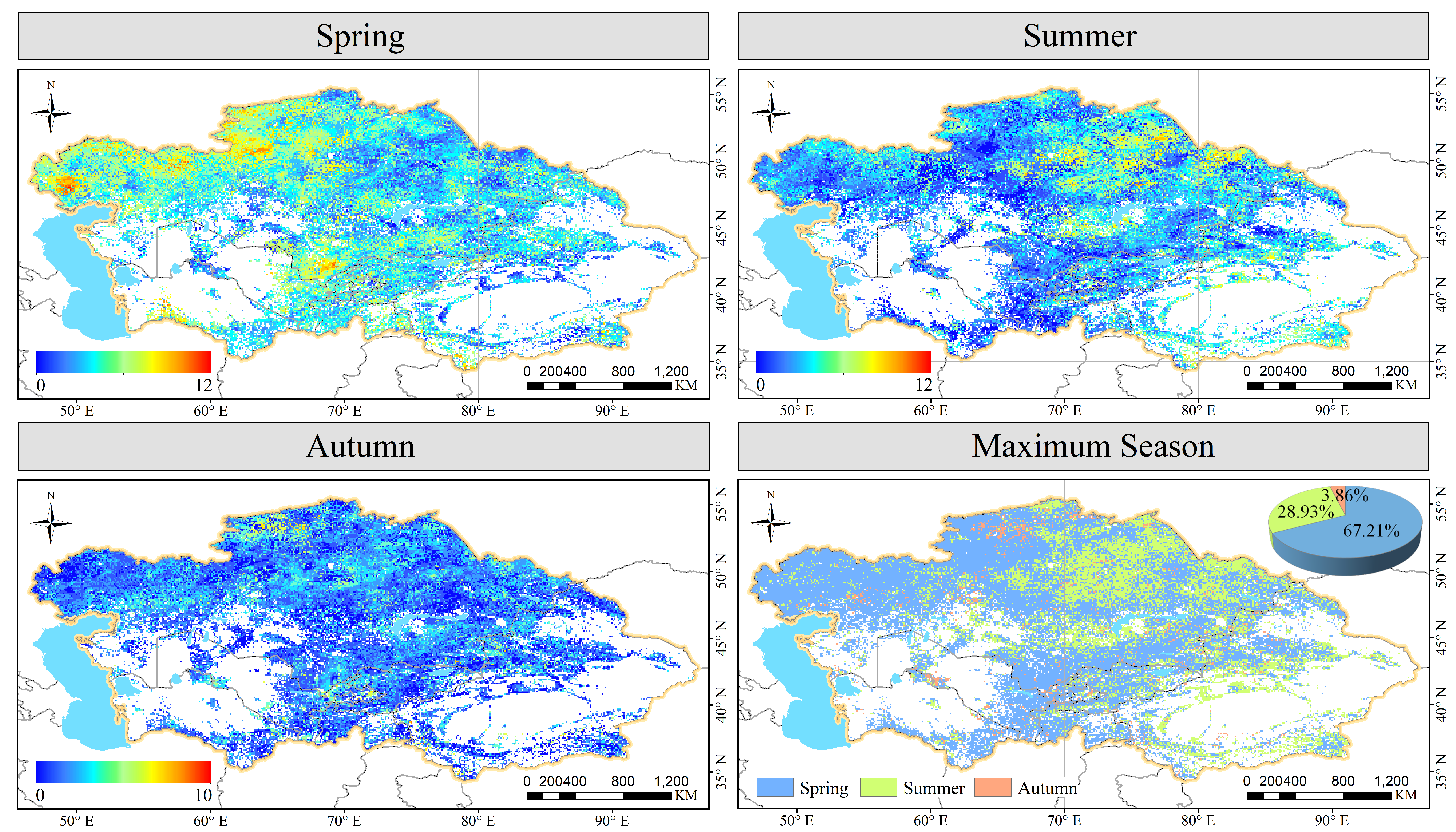

Supplement: S4 Fig — (TIF) [file pone.0352937.s005.tif]

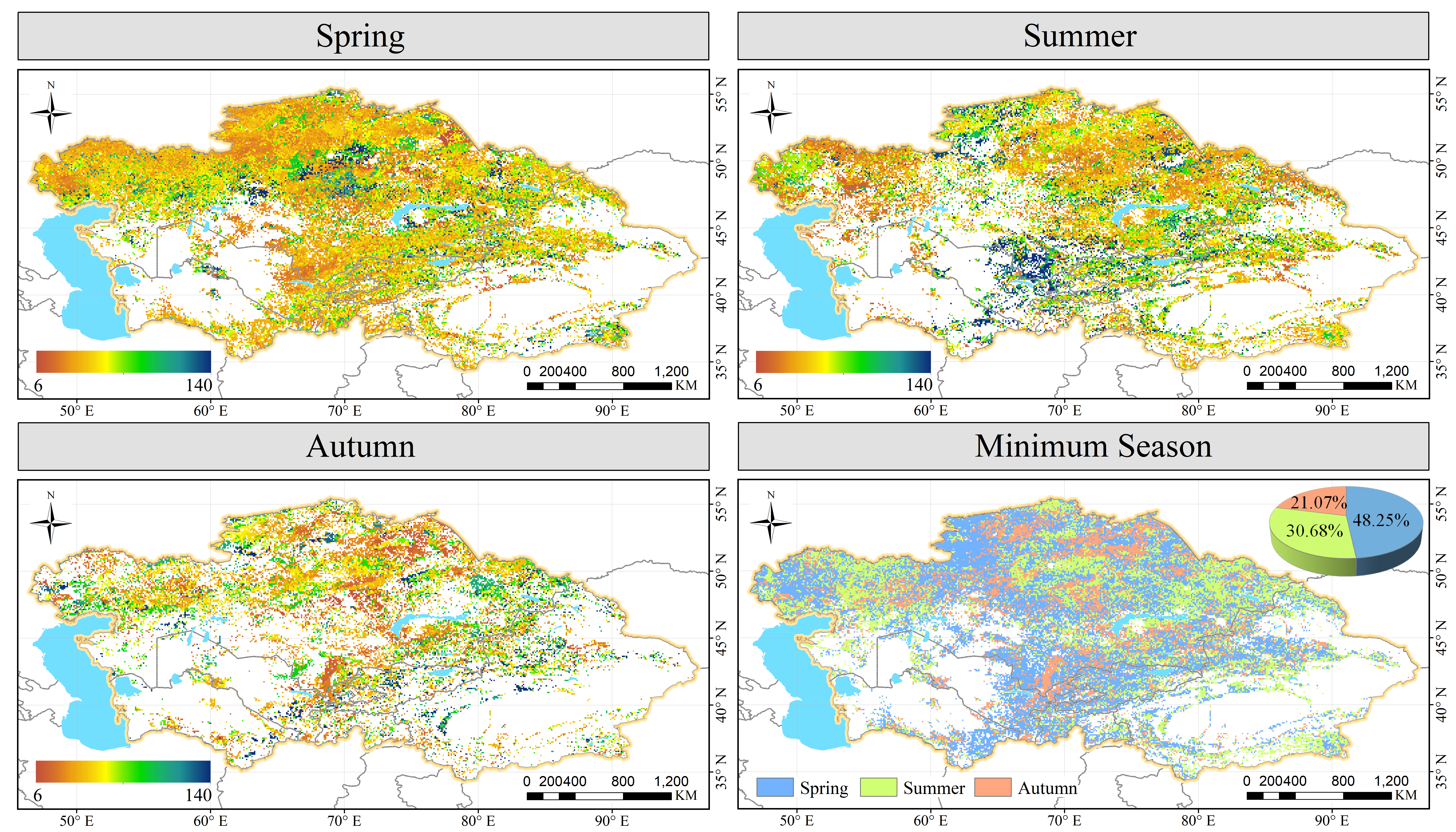

Supplement: S5 Fig — (TIF) [file pone.0352937.s006.tif]

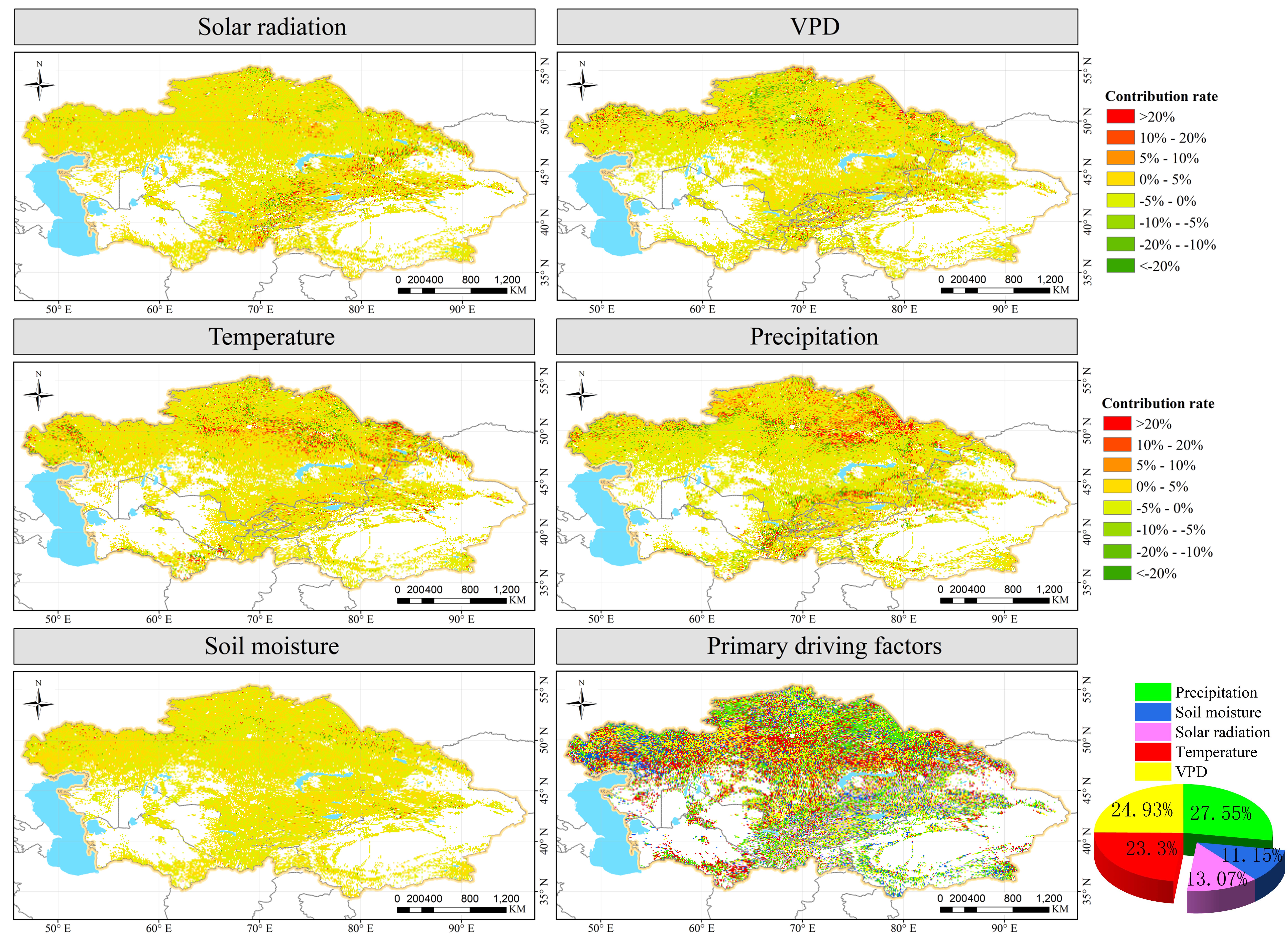

Supplement: S6 Fig — (JPG) [file pone.0352937.s007.jpg]

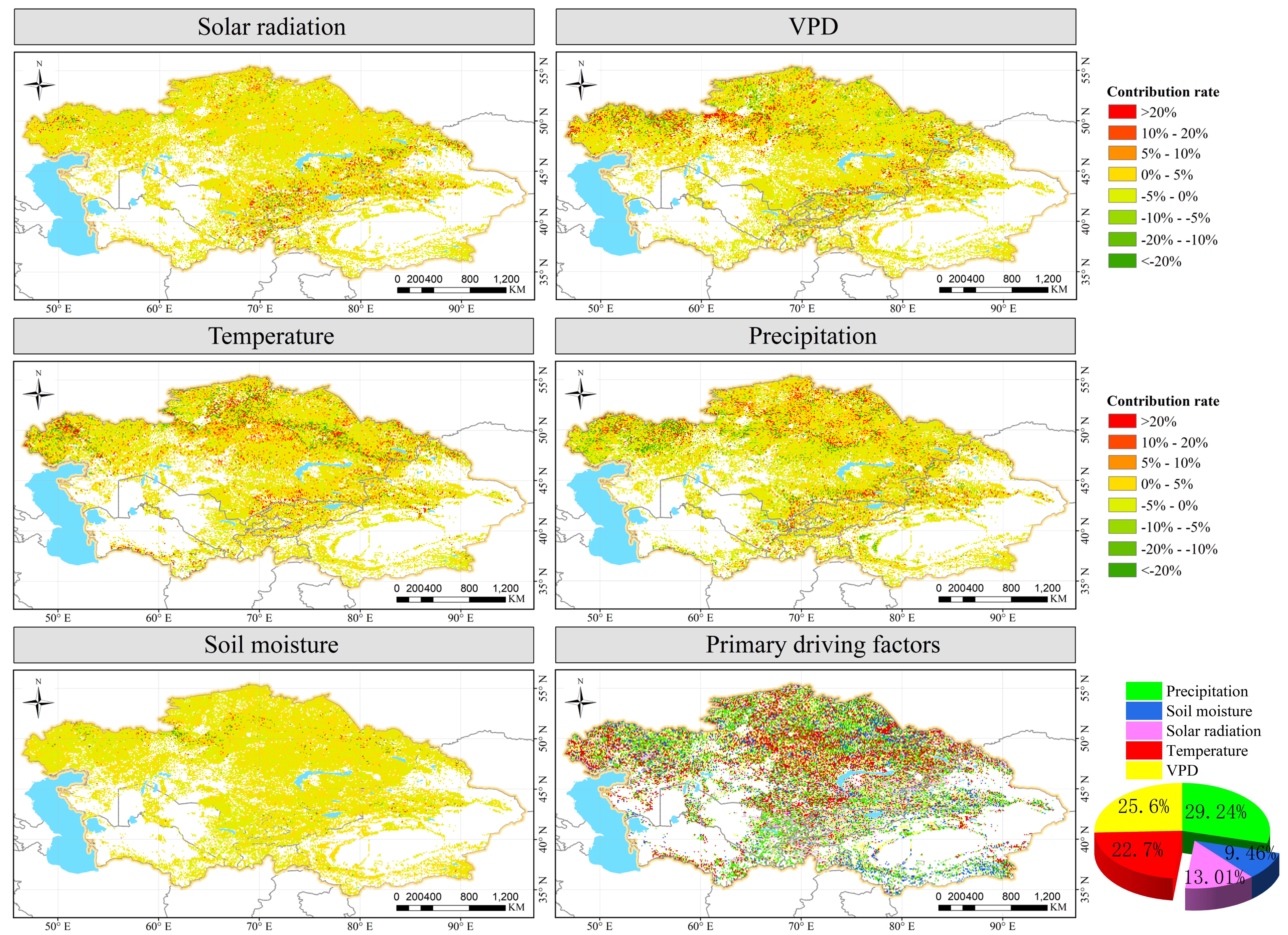

Supplement: S7 Fig — (JPG) [file pone.0352937.s008.jpg]

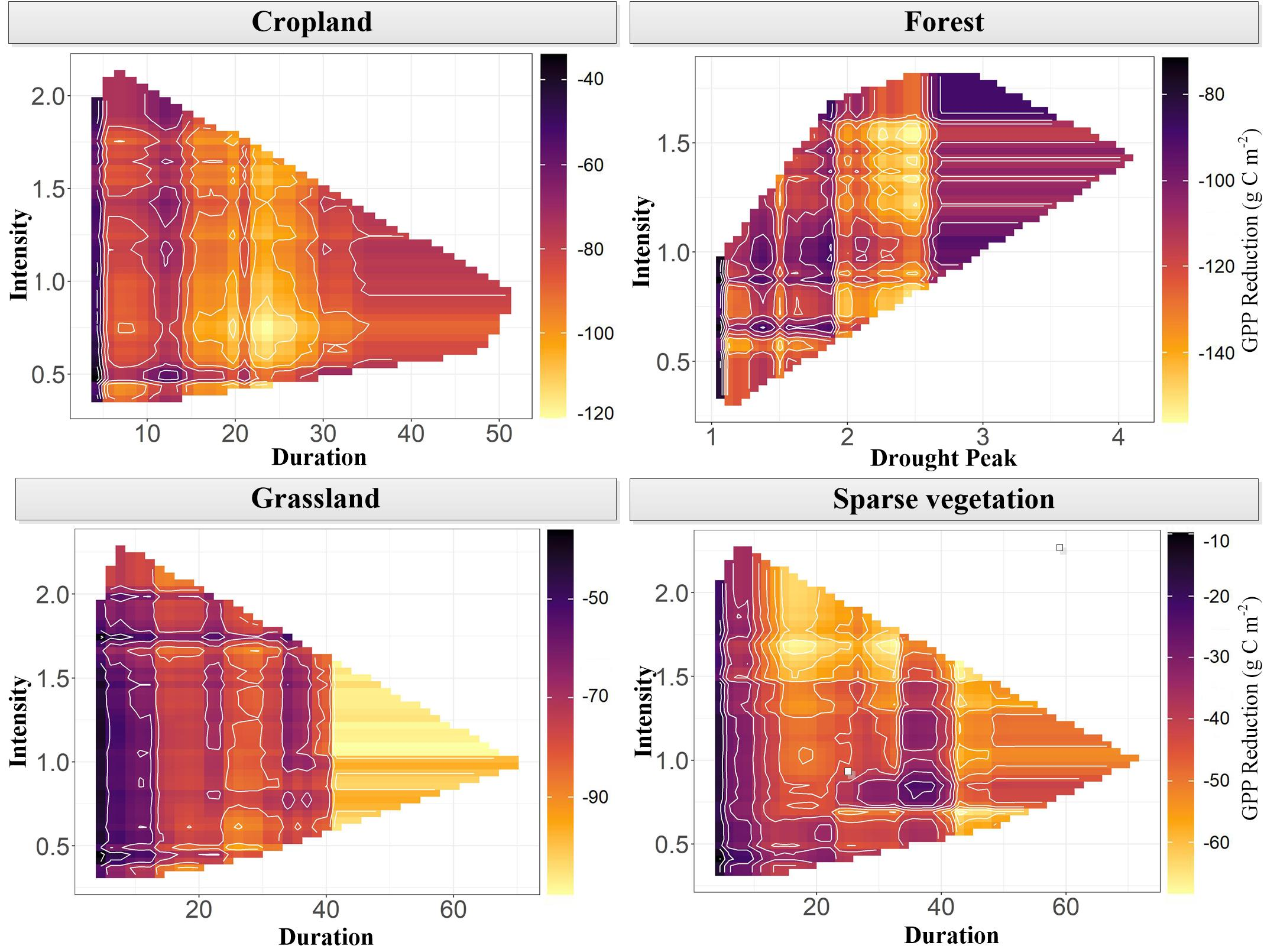

Supplement: S8 Fig — (TIF) [file pone.0352937.s009.tif]

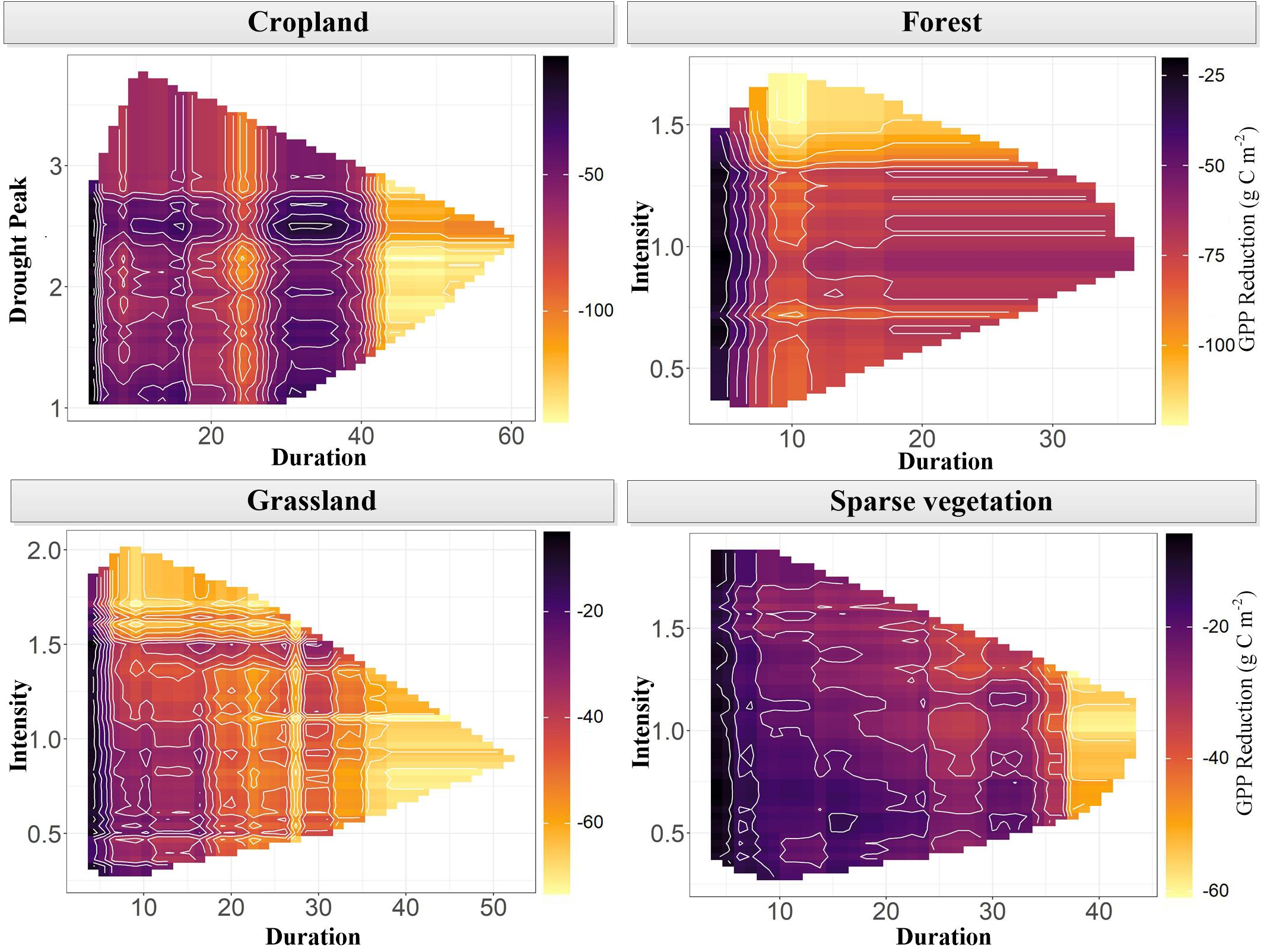

Supplement: S9 Fig — (TIF) [file pone.0352937.s010.tif]
