# Supplementary material for: Seasonal patterns of vegetation drought resilience and vegetation loss in Central Asia
Source: PLoS One. 2026 Jul 2;21(7):e0352937. doi: 10.1371/journal.pone.0352937 (PMC13327245; doi:10.1371/journal.pone.0352937)
Supplement: S1 Table — (DOCX) [file pone.0352937.s011.docx]

*Supplementary Information*

“Seasonal Patterns of Vegetation Drought Resilience and Vegetation Loss in Central Asia”

Liangliang Jiang ^1,^ ^2, 3*^, Guangming Wu ^2, 3^, Xinyuan Gui ^2, 3^, Xiaoran Liu ^1^

^1^ Chongqing Institute of Meteorological Sciences, Chongqing, China

^2^ School of Geography and Tourism, Chongqing Normal University, Chongqing, China

^3^ Chongqing Key Laboratory of GIS Application, Chongqing, China

**Table. S1 Collinearity test for drought characteristics.**

| **Characteristics** | **Spring** | | **Summer** | | | **Autumn** | | |  |
| --- | --- | --- | --- | --- | --- | --- | --- | --- | --- |
|  | **Tolerance** | **VIF** |  | **Tolerance** | **VIF** |  | **Tolerance** | **VIF** |  |
| Intensity | 0.22 | 4.46 |  | 0.28 | 3.57 |  | 0.18 | 5.71 |  |
| Severity | 0.03 | 28.71 |  | 0.04 | 24.23 |  | 0.04 | 24.74 |  |
| Peak | 0.47 | 2.15 |  | 0.46 | 2.19 |  | 0.41 | 2.43 |  |
| Interval | 0.30 | 3.30 |  | 0.25 | 4.02 |  | 0.30 | 3.28 |  |
| Times | 0.98 | 1.02 |  | 0.87 | 1.15 |  | 0.95 | 1.05 |  |
| Duration | 0.04 | 27.72 |  | 0.04 | 25.66 |  | 0.05 | 21.83 |  |
