# Supplementary material for: Seasonal patterns of vegetation drought resilience and vegetation loss in Central Asia
Source: PLoS One. 2026 Jul 2;21(7):e0352937. doi: 10.1371/journal.pone.0352937 (PMC13327245; doi:10.1371/journal.pone.0352937)
Supplement: S2 Table — (DOCX) [file pone.0352937.s012.docx]

*Supplementary Information*

“Seasonal Patterns of Vegetation Drought Resilience and Vegetation Loss in Central Asia”

Liangliang Jiang ^1,^ ^2, 3*^, Guangming Wu ^2, 3^, Xinyuan Gui ^2, 3^, Xiaoran Liu ^1^

^1^ Chongqing Institute of Meteorological Sciences, Chongqing, China

^2^ School of Geography and Tourism, Chongqing Normal University, Chongqing, China

^3^ Chongqing Key Laboratory of GIS Application, Chongqing, China

**Table. S2 Collinearity test for drought characteristics after removing drought severity.**

| **Characteristics** | **Spring** | | **Summer** | | | **Autumn** | | |
| --- | --- | --- | --- | --- | --- | --- | --- | --- |
|  | **Tolerance** | **VIF** |  | **Tolerance** | **VIF** |  | **Tolerance** | **VIF** |
| Intensity | 0.51 | 1.96 |  | 0.52 | 1.93 |  | 0.46 | 2.19 |
| Peak | 0.47 | 2.15 |  | 0.46 | 2.18 |  | 0.41 | 2.43 |
| Interval | 0.30 | 3.30 |  | 0.25 | 4.01 |  | 0.30 | 3.28 |
| Times | 0.98 | 1.02 |  | 0.88 | 1.14 |  | 0.95 | 1.05 |
| Duration | 0.28 | 3.56 |  | 0.23 | 4.26 |  | 0.28 | 3.53 |
